# Antimicrobial Activity of a Cationic Guanidine Compound against Two Pathogenic Oral Bacteria

**DOI:** 10.1155/2017/5924717

**Published:** 2017-05-04

**Authors:** E. Escamilla-García, A. G. Alcázar-Pizaña, J. C. Segoviano-Ramírez, C. Del Angel-Mosqueda, A. P. López-Lozano, E. Cárdenas-Estrada, M. A. De La Garza-Ramos, C. E. Medina-De La Garza, M. Márquez

**Affiliations:** ^1^Facultad de Odontología, Universidad Autónoma de Nuevo León, Monterrey, NL, Mexico; ^2^Centro de Investigación y Desarrollo en Ciencias de la Salud (CIDICS), Universidad Autónoma de Nuevo León, Ave. Gonzalitos s/n con Ave. Dr. Carlos Canseco, Mitras Centro, 64460 Monterrey, NL, Mexico; ^3^Facultad de Medicina, Universidad Autónoma de Nuevo León, Monterrey, NL, Mexico; ^4^Department of Oncology-Pathology, CCK, Karolinska Institutet, 171 76 Stockholm, Sweden

## Abstract

This study evaluated the potential antimicrobial properties of a polyguanidine (CatDex) on two oral bacteria. Chlorhexidine gluconate 1340 *μ*moL L^−1^ (CHX 0.12%) was used as control.* Streptococcus mutans (S. mutans)* and* Porphyromonas gingivalis (P. gingivalis)* were grown in BHI media. Bacterial sensitivity and antimicrobial activity were determined by the minimum inhibitory concentration (MIC) and Kirby-Bauer methods. To study side effects, that is, toxicity, dental pulp stem cells (DPSCs) were used. Fluorometric cytotoxicity and confocal microscopy assays were used in order to test cell viability. CatDex inhibited growth of* S. mutans *at all concentrations and growth of* P. gingivalis *at all concentrations except 25 *μ*moL L^−1^. The MIC of CatDex was 50 *μ*moL L^−1^ for both* S. mutans *and* P. gingivalis*. The inhibition of bacteria exposed for 8 h at 50 *μ*moL L^−1^ of CatDex exhibited increased antimicrobial activity over time, with 91% inhibition in both bacteria. The antimicrobial activities of CatDex and CHX were similar when tested on two common bacteria. CatDex was significantly less toxic to DPSCs. CatDex toxicity depended on time and not on concentration. With regard to clinical relevance, CatDex may have potential as a novel antimicrobial agent. Further studies are in progress.

## 1. Introduction

**Figure 1:**
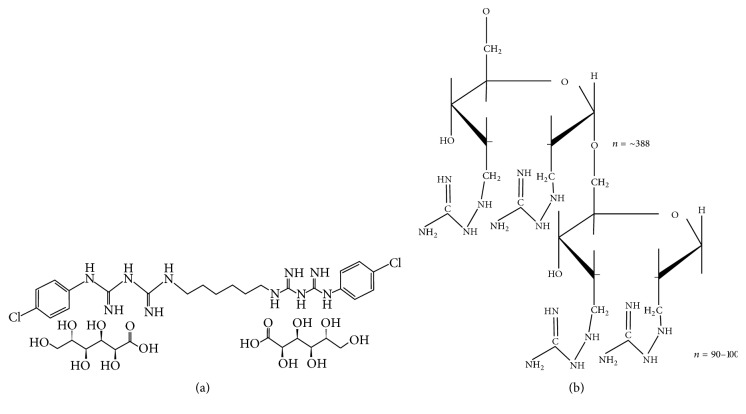
Chemical structure of chlorhexidine gluconate (CHX) (a) and CatDex (b).

It is estimated that over 90% of the world's population suffer or have suffered from some kind of oral/dental disorder, including periodontal disease and caries [1].* P. gingivalis* is considered to be the major etiological bacteria in the development of chronic periodontitis [2]. Dental caries is primarily caused by* S. mutans*. This and other bacteria can form biofilm or dental plaque [3]. Preventive measures against them include regular tooth brushing, flossing, fluoride therapy, fissure sealants, remineralisation of dental enamel, and antimicrobial agents [4]. Chlorhexidine gluconate (CHX) (Figure 1(a)) is used to prevent biofilm and remains the “gold standard” for oral antiseptics [5, 6]. CHX is a safe material with low potential toxicity when used correctly, although it may produce some undesirable side effects such as discolouration of dental enamel, pigmentation of anterior restorations, irritation of oral mucosa, and taste alteration. Moreover, CHX gluconate may not be suitable for application to mucous membranes [7]. Cases of allergic reactions have also been recorded [8, 9].

CHX cytotoxicity has been demonstrated in various cell lines [8]. It can induce apoptosis at low concentrations, while high concentrations result in cell necrosis [10, 11]. At certain concentrations, CHX appears to be toxic to human osteoblastic cells [12], odontoblast- like cells [13], and gingival fibroblasts. In addition, CHX may negatively affect wound healing [14].

CatDex is a polydisperse macromolecular construct with a molecular weight of 55 kD and a carbohydrate backbone with multiple covalently coupled guanidine side groups distributed along the carbohydrate chains (Figure 1(b)). It has a cationic electrostatic charge with a wide pH range and it is hydrophilic. CatDex demonstrates potent antitumour efficacy in several tumour cell lines [15, 16]. The proposed method of action in tumour cells is an electrostatic interaction with an anionic cell membrane, internalisation by the polyamine uptake system, and electrostatic binding of anionic structures in the cytoplasm, which kills the cell [15, 17]. Similar to CatDex, but as a hydrogel, cationic synthetic dextran demonstrated antimicrobial activity against* Escherichia coli* (ATCC 25922) and* Staphylococcus aureus* (ATCC 25923) [18].

There are several similarities in proliferation, growth, and progression between tumour cells and bacteria: one similar feature is the electrostatic condition of their cell wall/membrane. In neoplastic cells, there is an overexpression of N-acetylneuraminic acid (Neu5Gc); therefore, the cell membrane is negatively charged [19]. In bacteria, the electronegative charge of the cell wall is due to lipopolysaccharides in Gram-negative bacteria and teichoic acid in Gram-positive organisms. These similarities and differences between CHX and CatDex (Table 1) led us to consider conducting a study involving oral pathogens.

CatDex has demonstrated strong antitumour cell efficacy; it is for this reason that we decided to determine whether or not it showed antimicrobial activity, in addition to assessing its toxicity and safety. This study included the use of Gram-positive* S. mutans UA130* and Gram-negative* P. gingivalis W83*, two common oral bacteria associated with dental biofilm formation and periodontal disease.

## 2. Materials and Methods

### 2.1. Bacteria Culture

The strains used in this study were* Streptococcus mutans UA130* (ATCC700611) and* Porphyromonas gingivalis W83* (BAA308) as etiologic factors in periodontal disease and caries, the two most common oral diseases worldwide [1]. Culture and growth conditions for each bacterium were based on the technical specifications of the American Type Culture Collection (ATCC).* S. mutans* and* P. gingivalis* were subcultured at 37°C for 48 h on brain heart infusion agar plates (BHI, Becton Dickinson Bioxon®, Mexico). The bacteria were then inoculated to absorbance at 600 nm of 0.2 (Thermo Scientific GENESYS 10 UV Scanning Spectrophotometer, WI, USA) in Erlenmeyer flasks containing BHI medium.* S. mutans* and* P. gingivalis* were incubated for 6 and 23 h, respectively, at 37°C until the cultures reached late logarithmic growth (Thermo Scientific Lab-Line Incubator, USA). The strain* S. mutans* was cultured under aerobic conditions at 37°C.* P. gingivalis* was handled within an anaerobic chamber (Plas-Labs 855-ACB, Lansing, MI, USA), with an anaerobic atmosphere of H_2_ (10%), CO_2_ (5%), and N_2_ (85%) gas (Praxair, Mexico) at 37°C. Culture media and material were presterilised for 15 min at 120°C (All-American, Hillsville, USA). Before bacterial inoculation, the preculture was washed with 0.9% NaCl (w/v).

### 2.2. CatDex Conjugate Preparation and FITC Labelling

*CatDex Synthesis*. CatDex conjugate synthesis was performed as described previously [20]. Briefly, Dextran 70 Ph.Eur. (Pharmacosmos A/S, Denmark) was oxidised with sodium periodate and aminoguanidine (Sigma-Aldrich, Sweden) and subsequently conjugated. Sodium cyanoborohydride (Aldrich, Sweden), was used for reductive amination. Disposable PD-10 columns with Sephadex G-25 (GE Healthcare, UK) were used for separation and purification. The conjugation yield was determined by analysis of the total nitrogen content (by Mikro Kemi AB, Uppsala, Sweden, elemental analysis, method-MK2062).

Fluorescein isothiocyanate (FITC) labelling of CatDex was prepared as described by Márquez et al. [16]. In brief, 40 *μ*L FITC solution (50 mg, Sigma-Aldrich, Sweden) was mixed with 1 mL dextran conjugate (5 mg), all in 0.02 M borate buffer at pH 9.5. The solution was incubated overnight in a shaker in the dark and at room temperature and then purified on a PD-10 column equilibrated with PBS.

### 2.3. Evaluation of the Antimicrobial Activity of CatDex against Two Oral Bacteria

#### 2.3.1. Disk Diffusion Method

Disk diffusion method and the Kirby-Bauer method were used to test antimicrobial effects [21]. A bacterial culture was prepared under the same conditions as those indicated above until it reached the exponential growth phase. Then, 100 *μ*L of inoculum was expanded onto BHI agar plates. A filter paper disk (6 mm) (Cat. number 1440-185, Whatman, Piscataway, USA) was embedded in 20 *μ*L of CatDex solution (1, 5, 10, 25, 75, and 120 *μ*moL L^−1^) and then placed on the agar surface. The positive control was 0.12% (1340 *μ*moL L^−1^) chlorhexidine gluconate (CHX, Consepsis®, USA), commonly used in dentistry as a topical disinfectant in rinses and mouthwash [22]. Saline solution (0.9%) was used as a negative control. Culture plates were properly marked and incubated at 37°C for 24 to 48 h depending on bacteria growth requirements. Finally, the zone of inhibition around the disk was measured [23].

#### 2.3.2. Minimum Inhibitory Concentration (MIC)

The minimum inhibitory concentration method, considered the “gold standard,” was used to determine the susceptibility of microorganisms to antimicrobial compounds [23]. The concentration range for CatDex was determined from concentrations used by precedent studies in different tumour cell lines [16]. CHX was used as a positive control and saline solution served as a negative control.

*S. mutans *and* P. gingivalis *were previously cultivated as described above. A concentration between 10 × 10^7^ and 10 × 10^8^ cells/mL of both bacteria was inoculated in test tubes containing culture media and the testing compound was properly diluted to a final volume of 1 mL and incubated at 37°C for 24 h.

#### 2.3.3. Antimicrobial Effect of CatDex over Time

The antimicrobial effect of 50 *μ*moL L^−1^ (MIC value) CatDex on* S. mutans* and* P. gingivalis* was measured over 8 h. This procedure was performed in order to obtain information about antimicrobial behaviour. Test tubes were inoculated as discussed above and were brought to a final volume of 1 mL. Samples were incubated for 0 min and from 1 to 540 min. Saline solution was used as a negative control. Absorbance at 600 nm and pH values were measured after incubation.

### 2.4. Cytotoxicity Test

#### 2.4.1. Isolation and Culture of Dental Pulp Stem Cells (DPSCs)

Tooth collection and experiments were conducted with the approval of the Ethics Committee of the School of Dentistry, Universidad Autónoma de Nuevo León, Mexico, and signed patient consent was obtained (0041-SS-010618). Procedures were performed in accordance with the World Medical Association's Declaration of Helsinki of 1964 and subsequent revisions.

Dental pulp tissue was collected from human premolars and dissociated with 3 mg mL^−1^ collagenase type I and 4 mg mL^−1^ dispase (Sigma-Aldrich, USA) for 1 h at 37°C. The cell sample was centrifuged for 10 min at 300 g and filtered through a 70 *μ*m nylon filter (Millipore, Bedford, USA).

The DPSCs were cultivated for 3 weeks in *α*-modified Eagle's Medium (*α*-MEM) (Gibco, Invitrogen, Carlsbad, USA), containing 10% foetal bovine serum (FBS) and 1% antibiotic-antimycotic (Sigma-Aldrich). Cells were incubated at 37°C in a humidified atmosphere with 5% CO_2_ [24].

#### 2.4.2. Fluorometric Microculture Cytotoxicity Assay (FMCA)

This assay was performed as described by Larsson and Nygren [25]. The cytotoxicity of CatDex was tested on DPSCs cultivated in D-MEM containing 10% FBS and antibiotic-antimycotic. Briefly, 20 × 10^3^ cells per well were seeded into 96-well microtitre plates (Falcon, Becton Dickinson, France). CatDex was added at concentrations ranging from 10 to 120 *μ*moL L^−1^ and CHX was added at 1340 *μ*moL L^−1^ as a positive control; PBS was used as a negative control. After 24 h incubation, medium was removed by flicking the plates. Cells were washed three times with PBS. Fluorescein diacetate (FDA, Sigma) was dissolved in DMSO (Sigma-Aldrich) and kept frozen at −20°C as stock solution (10 mg mL^−1^). FDA was diluted in PBS at 10 *μ*g mL^−1^, and 200 *μ*L was added to each well. Plates were then incubated for 30 min at 37°C. A 96-well GloMax®-Multi+ Microplate Multimode scanning fluorometer (Promega, Madison, USA) was used at 495 nm. Data were analysed to determine cell viability (%).

#### 2.4.3. Cytotoxic Effect of CatDex over Time

The cytotoxicity test was performed as follows: 20 × 10^4^ cells per well were seeded into 96-well microtitre plates in media under conditions as described above for 24 h. Then CatDex (50 *μ*moL L^−1^ final concentration) was added and cells were incubated from 1 to 240 min (4 h), and the cytotoxic effect was measured by the FMCA method.

#### 2.4.4. Preparation of DPSCs Culture in a Coverglass System

DPSCs were seeded into eight wells in sterile chamber slides at 2 × 10^5^ cell well^−1^ (Chambered # German Coverglass System, Lab-Tek® II) with final volume of 400 *μ*L. A culture of DPSCs was made for 24 h as described above. Afterwards, CatDex or CHX was added with a final concentration of 50 *μ*mol L^−1^ and 550 *μ*mol L^−1^, respectively, that is, matching number of moles of each molecule. The cells were incubated for 1 and 5 h and MitoTracker® Red CM-H_2_XRos dye (300 nmol L^−1^) was added 30 min before the incubation time ended. Cells incubated with chlorhexidine at commercially used concentrations acted as a positive control and a negative control was provided by cells incubated in culture media without any other compound. After incubation, supernatant was removed and cells were fixed with 10% formaldehyde for 10 min and washed twice with PBS. Then 1 *μ*g mL^−1^ DAPI dye was added. Morphological analysis was performed by confocal laser microscopy (Axio Observer Z1/LSM 700, Zeiss) using Zen 2009 software and a 63x objective. MitoTracker, DAPI, and FITC dyes were excited with 561, 405, and 488 nm laser, respectively, at 2 mV.

### 2.5. Statistical Analysis

All experiments were performed in triplicate (*n* = 3). Mean values and standard deviation (SD) were calculated. Significant differences between CatDex and CHX were evaluated using Student's *t*-test (*p* ≤ 0.05).

## 3. Results

### 3.1. Sensitivity Test

**Figure 2:**
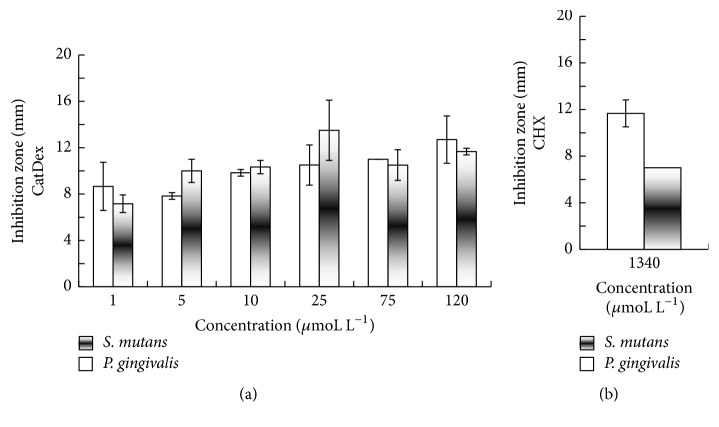
Determination of the bacterial susceptibility to CatDex (a) and CHX (b) of strains* S. mutans UA130* and* P. gingivalis W83*. Significant differences between CatDex and CHX were observed at all concentrations for both bacteria (*p* < 0.05) except at 1, 25, and 120 *μ*moL L^−1^.

The inhibitory effect of CatDex disk diffusion on both bacteria is shown in Figure 2. With CatDex, the mean zone of inhibition (SD) was 13.5 mm ± 2.59 at 25 *μ*moL L^−1^ for* S. mutans* and 12.7 mm ± 2.04 at 120 *μ*moL L^−1^ for* P. gingivalis *(Figure 2(a)). The mean results (SD) with CHX were 7 mm ± 0.00 and 11.7 mm ± 1.15 at 1340 *μ*moL L^−1^, respectively (Figure 2(b)).* S. mutans* was significantly more sensitive to CatDex than CHX at all tested concentrations (*p* ≤ 0.05). There was a significant difference between CatDex and CHX in* P. gingivalis* at 5 *μ*moL L^−1^ (*p* ≤ 0.05).

### 3.2. Minimum Inhibitory Concentration (MIC)

**Figure 3:**
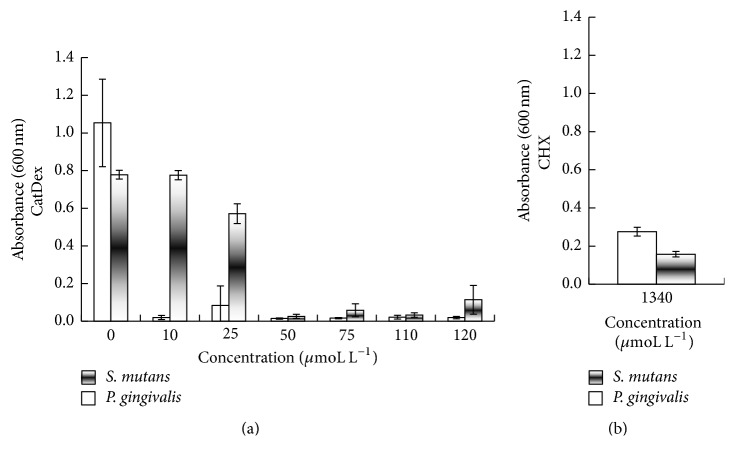
Determination of the minimum inhibitory concentration (MIC) of CatDex (a) and CHX (b) against* S. mutans UA130* and* P. gingivalis W83* (*p* ≤ 0.05). At all concentrations, CatDex showed a significant impact on* S. mutans*, and* P. gingivalis *growth also demonstrated a similar effect except at 25 *μ*moL L^−1^, both compared to CHX 1340 *μ*moL L^−1^ (*p* < 0.05).

The MIC was used to extend the results of the sensitivity test (Figure 3). Mean absorbance (*A*_600 nm_) (SD) of* S. mutans *and* P. gingivalis *cultures after 24 h of incubation at 37°C was 0.78 ± 0.02 and 1.15 ± 0.11, respectively. Final mean pH values (SD) were 5 ± 0.12 and 6 ± 0.32 for* S. mutans* and* P. gingivalis* (data not shown). CatDex showed ~100% bacterial inhibition at >50 *μ*moL L^−1^ (pH 7.1) for* S. mutans *and at >10 *μ*moL L^−1^ for* P. gingivalis* (Figure 3(a)). Bacterial inhibition with CHX at 1340 *μ*moL L^−1^ (Figure 3(b)) was 79% for* S. mutans *(pH 7.34). The effect on* P. gingivalis* was 76% inhibition (pH 7.41).

MIC results with CatDex and CHX showed significant differences at all concentrations for* S. mutans*. For* P. gingivalis*, there were significant differences at all concentrations except 25 *μ*moL L^−1^ (*p* ≤ 0.05).

### 3.3. Antimicrobial Effect of CatDex over Time

**Figure 4:**
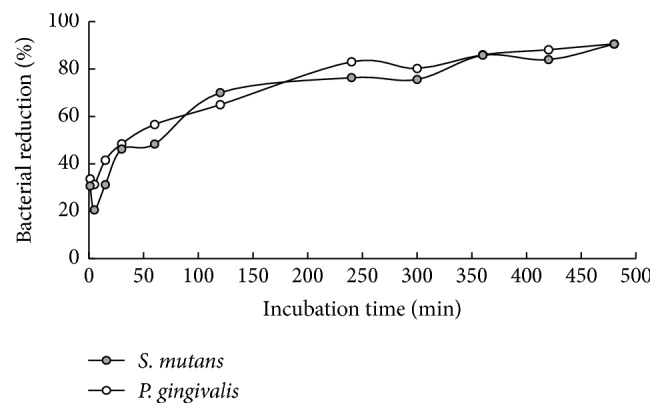
Antibacterial effect at 50 *μ*moL L^−1^ of CatDex. Percentage of bacterial reduction of* S. mutans UA130* and* P. gingivalis W83* over time (*p* ≤ 0.05).

CatDex was tested at 50 *μ*moL L^−1^ after MIC results in both bacteria (Figure 4). After the first minute, CatDex reduced the mean (SD) numbers of both bacteria by 31% ±  2.0 (pH 7.11). CatDex inhibition increased with time up to 91% at 480 min. CatDex showed a higher effect on* P. gingivalis *at 240 min (4 h) than on* S. mutans*.

### 3.4. CatDex Cytotoxicity Evaluation

**Figure 5:**
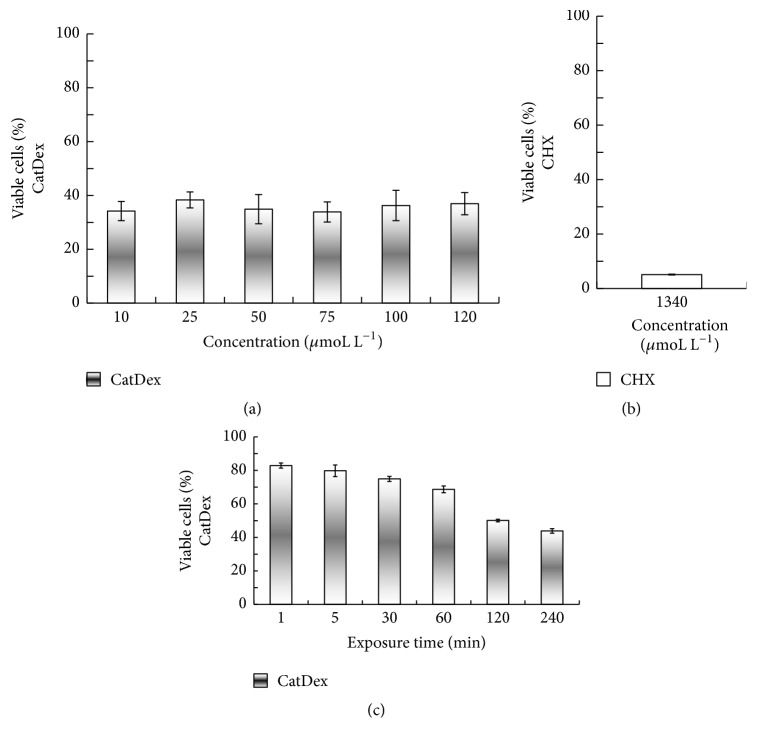
Cytotoxic effect at different concentrations of CatDex and CHX for 24 h at 50 *μ*moL L^−1^ (a) and 1340 *μ*moL L^−1^ (b), respectively, and for 240 min (c) on dental pulp stem cells (DPSCs). No correlation was found between viable cell percentage and CatDex concentration (*p* < 0.05).

The CatDex cytotoxicity test results are shown in Figure 5. The mean (SD) viability of DPSCs after 24 h of exposure to CatDex ranged from 34% ± 3.70 to 38% ± 2.96 at concentrations between 10 and 120 *μ*moL L^−1^, respectively, as shown in Figure 5(a). The mean (SD) viability of DPSCs after 24 h of exposure to CHX at 1340 *μ*moL L^−1^ was 5.01 ± 0.157 (Figure 5(b)).

The MIC concentrations of CatDex (50 *μ*moL L^−1^) for* S. mutans *and* P. gingivalis *were tested at different incubation times (see Figure 5(c)). In the first minute, we observed mean (SD) cell viability of 83% ± 1.53. The mean (SD) viability decreased gradually with time of exposure: from 80% ± 3.51 at 5 min to 44% ±  1.32 at 240 min of exposure to CatDex, with 50% ± 0.76 and 44% ± 1.32 of viability for* S. mutans* and* P. gingivalis*, respectively.

### 3.5. Cytotoxic Effect and Morphological Changes on DPSCs

**Figure 6:**
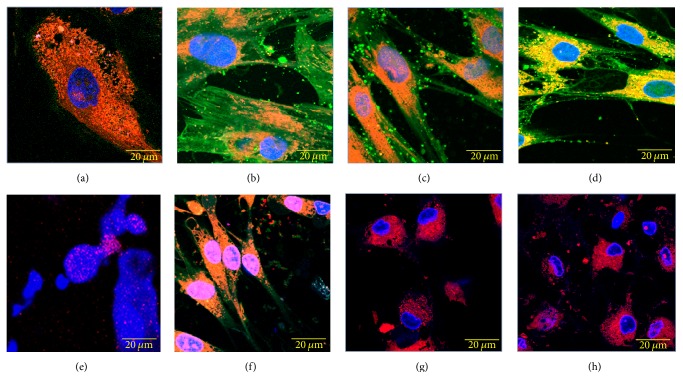
Cytotoxic effect on cultured dental pulp stem cells (DPSCs) exposed to 50 *μ*moL L^−1^ CatDex-FITC and CHX for different amounts of time. (a) Negative control; (e) positive control. (b), (c), and (d): CatDex-FITC at 3 sec, 1 h, and 5 h, respectively. (f), (g), and (h): chlorhexidine at 3 sec, 1 h, and 5 h, respectively. In red, emission of MitoTracker-H_2_XRos staining mitochondria; in blue, DAPI signal localised to the nuclear compartment; in green, CatDex-FITC signal localised to cytoplasm; and in orange, colocalisation of MitoTracker and FITC signals. Confocal laser microscope, fluorescent, histochemical technique, and objective 63x.

Figure 6 shows the effect on viability of DPSCs exposed to CatDex-FITC (0.05 *μ*mol L^−1^) for 0, 1, and 5 h. In the negative control (Figure 6(a)), cellular morphology is normal, with sizes between 50 and 100 *μ*m in their major diameter and between 10 and 40 *μ*m in their minor diameter; they exhibit adherent cytoplasm extensions that end in thin threadlike processes, which lends a starry appearance to the cell. The nucleus was round or oval with a smooth surface and a diameter of about 20 × 30 *μ*m. Dispersed chromatin in blue by DAPI staining was observed. Cytoplasm was starry irregular or fusiform. Abundant mitochondria profiles, circular, oval, or elongated, in red colour were seen by MitoTracker.

The positive control with CHX (Figure 6(e)) shows chromatin condensation, pyknosis, and nuclear disorganisation, along with mitochondrial disintegration, decreased MitoTracker signaling, and cytoplasmic disorganization, with MitoTracker discharge to culture medium. The effect of CatDex is shown in Figures 6(b), 6(c), and 6(d) at 0, 1, and 5 h of exposure, respectively. Cells retained normal morphological appearance (Figure 6(a)) independent of incubation time. Green fluorescence from FITC is observed in the cells' cytoplasm: this signal apparently decreased over time. MitoTracker signal was observed in the perinuclear region of the cytoplasm and is colocalised with the green signal of FITC.

Contrast images from Figures 6(f), 6(g), and 6(h) describe the effect on similar cultures of CHX over time. At time zero (Figure 6(f)), cells showed a morphological appearance similar to that of the positive control. After 1 and 5 h of exposure to CHX, the cells showed a cytoplasm of circular appearance and decreased membrane extensions, in addition to an irregular surface and a heterogeneous texture with round red agglomerates of variable size. The nucleus is decreased in size with folding in its surface and condensed chromatin, which gives a pyknotic appearance. This change is more pronounced at 5 h of exposure.

## 4. Discussion

We tested the possible antimicrobial properties of CatDex against the bacteria* S. mutans* and* P. gingivalis* and its toxicity towards DPSCs. CatDex demonstrated antimicrobial effects against these two bacteria, especially* P. gingivalis*. CHX is a commonly used antiseptic with broad-spectrum activity against a large number of oral microorganisms. It is less effective against Gram-negative microorganisms due to the lipopolysaccharides (LPS) found in the cell membrane of these microorganisms [26].

CHX is an agent with multiple amine and imine groups. It contains a cationic charge that interacts electrostatically with anionic structures of the bacterial membrane wall. CHX destabilises the cell wall and interferes with osmosis; this mechanism of action is found in many cationic agents [7, 27]. CatDex is a polyguanidine compound that is more stable than CHX and has a strong cationic electrostatic charge at a broad pH interval. CatDex and CHX both have a cationically charged molecule that binds anionic groups and it is reasonable to assume that they act upon bacteria in a similar way. It is well known that CHX is bacteriostatic at low concentrations and bactericidal at high concentrations and that it is less effective against Gram-negative organisms [28]. CHX is more effective at an alkaline pH and its activity is greatly reduced in the presence of organic matter [29]. At high temperatures, CHX decomposes into chloroaniline, which may explain its maximal action only in the first minutes of contact with bacteria [30].

Antibiotics and other antibacterial substances do not easily penetrate the outer membrane of Gram-negative bacteria because of their hydrophobic components. Antibiotics, which are active against Gram-positive bacteria, are often much less active against Gram-negative bacteria [26]. CatDex showed an inhibitory effect on bacterial growth which was dependent on concentration and exposure time. CatDex showed gradual and sustained inhibition for up to 8 h for both bacteria, which can be explained by the high stability of the CatDex molecule.

With regard to cytotoxicity, CHX has been shown to be less toxic to fibroblasts and keratinocytes compared to H_2_O_2_ and NaClO [31], and its toxic potency is dependent on length of exposure and medium composition [32]. Most studies have shown that CHX tends to damage different cell lines such as osteoblastic, endothelial, and fibroblastic cells [33]; furthermore, a recent study demonstrated CHX's toxic effect on stem cells from human exfoliated deciduous teeth at similar therapeutic concentrations over different periods of time [34]. Our results showed CatDex to be significantly less toxic to DPSCs than CHX, with a comparable antimicrobial effect towards both bacteria.

Cytotoxicity on DPSCs was different between CatDex and CHX even after 5 h of exposure: morphologically, the integrity of DPSCs exposed to CatDex was maintained, whereas CHX caused evident cellular damage. We concluded that CHX is significantly more toxic than CatDex.

In the search for an ideal endodontic irrigant with the four major desirable properties (antimicrobial activity, nontoxicity, water solubility, and capacity to dissolve organic matter), our results and literature [20] show that CatDex more than meets most of these criteria.

## 5. Conclusion

CatDex has an antimicrobial effect on* S. mutans *and* P. gingivalis *similar to that of CHX. CHX cell toxicity was dependent on concentration and time, while CatDex toxicity depended only on time. CatDex was less toxic to DPSCs over long exposure times and did not alter cell morphology. With the growing evidence of the potential involvement of oral bacteria in the pathogenesis of upper digestive tract neoplasia [35], studies of new antibacterial compounds, even with known antitumour agents such as CatDex, are further warranted.

## Figures and Tables

**Table 1:** Comparative table of the physicochemical and biological properties of CatDex and CHX.

Characteristics	CHX	CatDex
	Gold standard antiseptic	?

Solubility	Hydrophilic	Hydrophilic

Charge	Cationic polybiguanide (polybiguanide)	Cationic

Molecular weight	1340 g moL^−1^	55 KD

Density	1.01 g cc^−1^	?

pH	5.5–7.5	6.5

Appearance	Blue translucent liquid	Amber translucent liquid

Activity	Bactericide Fungicide Antiviral	Antitumour efficacy [15, 16]

Interaction with cells	Electrostatic interaction with cationic lipopolysaccharides and teichoic acid of cell wall [19]	Electrostatic interaction with anionic structures of cytoplasm [15, 17]
